# Exercise Enhances Hippocampal Recovery following Binge Ethanol Exposure

**DOI:** 10.1371/journal.pone.0076644

**Published:** 2013-09-30

**Authors:** Mark E. Maynard, J. Leigh Leasure

**Affiliations:** 1 Department of Psychology, University of Houston, Houston, Texas, United States of America; 2 Department of Biology & Biochemistry, University of Houston, Houston, Texas, United States of America; University of Victoria, Canada

## Abstract

Binge drinking damages the brain, and although a significant amount of recovery occurs with abstinence, there is a need for effective strategies to maximize neurorestoration. In contrast to binge drinking, exercise promotes brain health, so the present study assessed whether it could counteract ethanol-induced damage by augmenting natural self-repair processes following one or more binge exposures. Adult female rats were exposed to 0 (control), 1 or 2 binges, using an established 4-day model of binge-induced neurodegeneration. Half of the animals in each group remained sedentary, or had running wheel access beginning 7 days after the final binge, and were sacrificed 28 days later. To assess binge-induced hippocampal damage and exercise restoration, we quantified volume of the dentate gyrus and number of granule neurons. We found that a single binge exposure significantly decreased the volume of the dentate gyrus and number of granule neurons. A second binge did not exacerbate the damage. Exercise completely restored baseline volume and granule neuron numbers. To investigate a potential mechanism of this restoration, we administered IdU (a thymidine analog) in order to label cells generated after the first binge. Previous studies have shown that neurogenesis in the dentate gyrus is decreased by binge alcohol exposure, and that the hippocampus responds to this insult by increasing cell genesis during abstinence. We found increased IdU labeling in binge-exposed animals, and a further increase in binged animals that exercised. Our results indicate that exercise reverses long-lasting hippocampal damage by augmenting natural self-repair processes.

## Introduction

Binge drinking is a common alcohol use disorder (AUD) in the United States – one in six American adults reports binge drinking four times a month (eight drinks per binge) [1]. Binge drinking damages fronto-temporal brain regions important for memory, decision-making and behavioral control [2], [3], thereby perpetuating intake by limiting the cognitive control necessary for cessation [4], [5]. It is also a risk factor for stroke [6] and dementia [7], [8].

With abstinence from alcohol, natural healing occurs in the brain [9]–[12], but much remains to be learned about this self-repair. Animal models of binge alcohol consumption replicate the pattern of brain damage seen in binge drinking humans [13]–[16], and provide a means by which to systematically study the mechanisms of alcohol-induced neuronal damage and endogenous self-repair processes, as well as test potential therapeutic interventions. Such studies show that binge alcohol exposure kills cells [13] and also decreases neurogenesis in the dentate gyrus (DG), a neurogenic region of the adult central nervous system [17], [18]. These combined effects of cell death and decreased cell birth are associated with a significant loss of DG granule neurons immediately following a single binge exposure [19]. In response to binge alcohol exposure, the DG undergoes two distinct reactive bursts of cell proliferation [18], [20]. The second burst, which occurs 7 days post-binge, results in a significant increase in new neurons, which may serve to repopulate the depleted granule cell layer [18].

Binge drinking is a pattern of intake and by definition occurs more than once. It has been suggested that the repeated cycles of intoxication and abstinence that characterize binge drinking may be particularly damaging to the brain [21]–[23]. This may be due in part to disruption of on-going post-binge repair processes by subsequent binge episodes, which could suppress repair attempts while simultaneously causing further damage. In the present study, we tested this hypothesis by exposing animals to a second binge 6 days after the first, in order to determine whether it would disrupt the reactive cell genesis previously shown to occur on post-binge day 7. We assessed survival of cells born on day 7 post-binge and quantified the volume of the DG and number of remaining granule neurons 28 days later. We hypothesized that a second binge exposure would disrupt cell genesis after the initial binge, and that this disruption would be manifested by decreased DG volume and loss of granule neurons.

A wealth of prior research indicates that post-injury neural events can be influenced by behavior (for reviews see [24]–[27]). One such behavior is exercise - a powerful promoter of neuroplasticity, which may prove efficacious for augmenting post-binge brain repair. It increases neurogenesis in the DG [28], [29], and could therefore enhance the reactive cell generation that occurs post-binge. Moreover, it has been shown to help the brain recover from developmental alcohol exposure [30]–[32], as well as protect it from a subsequent binge [19]. In the present study, animals exercised for 28 days, beginning 7 days after a single or second binge exposure. We hypothesized that exercise would augment survival of cells generated in the DG post-binge, thereby normalizing both DG volume and number of granule neurons.

## Materials and Methods

### Ethics Statement

All experimental procedures were conducted in accordance with the Guide for the Care and Use of Laboratory Animals of the National Institutes of Health. The relevant animal protocol was approved by the University of Houston Institutional Animal Care and Use Committee (protocol number 11-021).

### Animals and housing conditions

**Figure 1:**
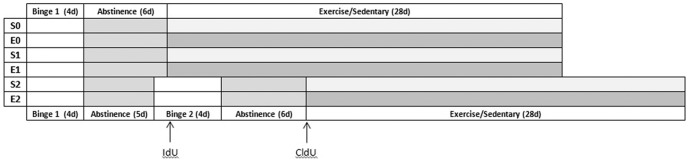
Experimental design and time course of events. Animals underwent either 0 (control diet), 1 or 2 binges. Beginning 7 days after the last binge, half the animals in each group exercised for 28 days (groups E0, E1, E2). In order to label cells generated in response to the first binge, all animals received IdU 7 days after the last dose of ethanol. In order to label cells generated in response to the second binge, animals in the Two Binge groups (S2 and E2) received CldU 7 days after the end of the second binge. All animals were sacrificed 35 days after the last binge.

Figure 1 shows the experimental groups and timeline of this study. Forty-eight female Long-Evans rats weighing 175 to 200 grams and aged two months at the beginning of the experiment were randomly divided into 6 groups in a 3×2 design comparing Diet (Control, Single Binge, Two Binge) and Activity (Sedentary or Exercise). Each group consisted of 8 animals: Sedentary Controls (S0) were sedentary and received an isocaloric control diet; Sedentary Single Binge (S1) animals were sedentary and received ethanol diet over one four-day binge; Sedentary Two Binge (S2) animals were sedentary and received the ethanol diet over two four-day binge periods; Exercise Controls (E0) received isocaloric control diet and later exercise access; Exercise Single Binge (E1) rats received the ethanol diet over one four-day binge and later had exercise access; and Exercise Two Binge (E2) animals, which received the ethanol diet over two four-day binge periods and later had exercise access. Animals were group housed in clear Plexiglas cages on a reversed light/dark cycle (lights off at 9:00/on at 21:00), with *ad libitum* rat chow and water. Prior to beginning the experiments, all rats were tamed by gentle handling to acclimate them to the experimenters and make them amenable to gavage. Female rats were chosen because of their consistent running behavior and because they have not been studied extensively in this model of an AUD.

### Binge paradigm

During binge exposure, food was removed from both control and binged animals, but water was always available. Ethanol was administered via intragastric gavage according to a previously used paradigm [13], [14], [17]–[19] modified from Majchrowicz (1975). Rats were gavaged with ethanol diet (25% ethanol w/v in vanilla Ensure™; Abbot Laboratories, Columbus, OH) or isocaloric control diet (dextrose w/vanilla Ensure™) every 8 hours for 4 days, starting on the first day of the experiment (12 doses total). The initial dose for each animal was 5 g/kg and caused significant intoxication; further doses were determined based on a 6-point behavioral intoxication scale (0 =  normal; 1 = hypoactive; 2 = ataxia; 3 =  ataxia+dragging abdomen and/or delayed righting reflex; 4 = absent righting reflex; 5 = absent eye blink reflex). Each point on the scale corresponds to an accompanying dose of ethanol, such that the greater the observed behavioral intoxication, the smaller the subsequent dose. Animals in the two binge exposure groups began their second binge 6 days after their last dose of alcohol (see Figure 1).

### Blood ethanol concentration

Blood ethanol concentration (BEC) was determined from tail blood samples taken 90 minutes after the morning dose on day 3. Samples were centrifuged, and then stored at −20°C until further analysis. Serum was extracted and BEC determined using a GM7 Analyzer based on external standards (Analox, MA, USA).

### Monitoring of withdrawal symptoms

Eight hours after the last dose of ethanol, food was replaced in the animals' home cages. Spontaneous withdrawal behavior in all ethanol treated rats was monitored for hours 10–26 after the last dose. This range corresponds to the peak period of withdrawal [33]. Rats were observed in their home cages and scored for spontaneous withdrawal behaviors in 30-minute intervals. Red lamps were used during the dark cycle so as to not disturb Circadian rhythms. Behaviors were scored based on the withdrawal scale of Penland and colleagues [34]. At every time point, the spontaneous withdrawal symptoms of each animal were observed and the most severe symptom recorded. Mean withdrawal severity and peak withdrawal severity scores were calculated. The mean withdrawal score refers to the average of the scores observed, and peak withdrawal score refers to the average of the most severe withdrawal symptom observed in each animal during the entire 17-hour period.

### Estrus Cycle Monitoring

Vaginal smears were taken once each day between 8:00 and 9:00 A.M. Each sample was placed onto a slide, stained with cresyl violet and coverslipped. Samples were viewed under a light microscope at 10× magnification and the determination of the stage of estrus (proestrus, estrus, metestrus, diestrus) was made.

### Administration of thymidine analogs

In order to label cells generated in response to the first binge, all animals were injected with iododeoxyuridine (IdU, MP Biomedicals, Ohio, USA, 300 mg/kg, i.p.), a thymidine analog, seven days following the first binge episode (see Figure 1). To detect cells born in response to a second binge, a different thymidine analog, chlorodeoxyuridine (CldU, Sigma, MO, USA, 300 mg/kg, i.p.), was administered to all Two Binge animals seven days following the second binge.

### Voluntary exercise

On the seventh day following the last dose of alcohol or isocaloric diet, rats in the exercise groups (E0, E1, E2) were given access to exercise wheels for a maximum of five and a half hours daily, for four weeks. Daily exercise access began at the onset of the dark cycle. The 4-weeks of daily exercise was begun on the seventh post-binge day for two reasons. First, binge alcohol exposure damages the brain, and the initial seven days following brain injury is a vulnerable period during which increased activity can exacerbate damage and limit recovery [35], [36]. Second, we wanted to augment the burst of cell proliferation that has been shown to occur on post-binge day 7 in this model [18]. In order to precisely monitor distance travelled, rats were removed from home cages and placed into individual running wheels equipped with counters. During the exercise period, animals had access to food and water *ad libitum*. After exercise, animals were returned to their home cages. Sedentary animals remained in their home cages.

### Histology

After four weeks of exercise (35 days after the last dose of alcohol) each rat was given an overdose of anesthetic and intracardially perfused with cold saline, followed by 4% paraformaldehyde until the upper body was stiff. Brains were removed and post-fixed overnight, and then refrigerated in 30% sucrose. Brains were cut in 50 µm coronal sections on a freezing microtome. Sections were stored in cryprotectant in 96-well microtiter plates at −20°C until further processing.

For immunohistochemistry, sections were quenched for 10 minutes at room temperature in 0.6% hydrogen peroxide (to exhaust the activity of endogenous peroxidases) followed by six 10-minute washes in TBS. These sections were then incubated for 10 minutes in 2N HCl at 37°C, followed by a 10 minute rinse in 0.1 M borate buffer. Sections were then rinsed six times in fresh TBS for 10 min. IdU and CldU sections were then blocked for 60 minutes in 3% normal donkey serum (Sigma-Aldrich, MO, USA), followed by incubation at 4°C for 72 hours in primary antibody (IdU: mouse anti-BrdU, Becton Dickenson, NJ, USA; 1∶250; CldU: rat anti-BrdU, Accurate Chemical & Scientific Corporation, NY, USA; 1∶250). After two TBS rinses for 15 minutes each, sections were blocked with 3% donkey serum twice for 30 minutes each. The tissue was then incubated overnight at room temperature in secondary antibody (IdU: biotinylated donkey anti-mouse or CldU: biotinylated donkey anti-rat, both from Jackson ImmunoResearch Laboratories, PA, USA; 1∶250). Next, sections were rinsed three times in TBS for 15 minutes each, then treated for 60 minutes in avidin-biotin complex (ABC, Vector Labs, Burlingame, CA, USA) and then rinsed three times in TBS for 10 minutes each. Sections reacted and were visualized with diaminobenzidine (DAB) and were then rinsed four times in TBS for 10 minutes each, before being mounted onto gelatinized slides, counterstained with methyl green, and cover slipped using Protexx. Slides were then coded so that when tissue was viewed under the microscope, the investigator was blind to experimental condition.

### Stereology

The number of granule cells in the DG was determined using the optical fractionator method applied via an automated stereology system (StereoInvestigator, MicroBrightField, VT, USA). Using a Nikon Eclipse 80i upright microscope, the region of interest was traced using the 10× objective, and cells were counted within two-dimensional counting frames using a 100× oil objective. The average mounted section thickness was approximately 37 µm, thus top and bottom guard zones were set at 5 µm each, for an optical dissector height of 27 µm. Granule cells were counted in every sixth section in a single hemisphere beginning at the earliest emergence of the DG at Bregma −1.80 mm and ending at Bregma −6.04 [37]. This resulted in 10–12 sections per brain. The counting frame size was 40×40 µm and the grid size was 200×200 µm. The volume of the DG was determined in the same sections, using the Cavalieri estimator, applied via StereoInvestigator.

### Quantification of labeled cells

Cells labeled with IdU or CldU were quantified in separate series of sections. Each labeled soma in the granule cell layer or subgranular zone (defined as zero to two cell bodies from the inner molecular layer) was counted in every sixth section from Bregma −1.88 through Bregma −6.04 [37], using a 40× oil objective.

### Statistical analyses

All values presented are expressed as mean ± standard error of the mean. Body weight was analyzed using two-way repeated measures ANOVA and the variables Time, Activity, Diet, and their respective interactions. Running distance was analyzed using repeated measures ANOVA and the Time, Diet, and Time×Diet interaction. Behavioral intoxication, ethanol dose, and spontaneous withdrawal scores were analyzed using repeated measures ANOVAs and the Time, Binge, and Time×Binge interaction; for these analyses Binge 1 includes data from all alcohol treated animals during the first binge (S1, E1, S2, E2) and Binge 2 includes data from Two Binge animals during the second binge (S2, E2). Neuroanatomical data (granule cells, volume, and IdU+ cells) was analyzed with two-way factorial ANOVA using the variables Activity, Diet, and the Activity by Diet interaction. Planned Bonferroni-corrected *post hoc* comparisons were used when appropriate. All two-group comparisons between a single and two binge exposure, e.g. BECs (mg/dl), ethanol dose (g/kg), peak intoxication and peak withdrawal behaviors were analyzed with independent groups *t*-tests. An independent groups *t*-test was used to compare CldU+ cells in sedentary and exercised Two Binge animals. Paired groups *t*-tests were used to compare the number of IdU+ to CldU+ cells in sedentary and exercised Two Binge animals. Pearson correlations were performed in order to examine the relationship between behavioral intoxication and mean withdrawal score, between IdU+ cells and DG volume in binge-exposed animals and between IdU+ cells and number of granule neurons in binge-exposed animals. Significance of the correlations was determined using the critical value table for Pearson's Correlation Coefficient. For all statistical analyses, a *p*-value of less than 0.05 was deemed significant.

To determine whether the stage of estrus affected neuroanatomical outcomes, a factorial ANOVA using the variables Diet, Activity, and Day 1, 2, 3, and 4 of the First Binge was analyzed. Because stage of estrus is a categorical variable, it was necessary to dummy code by assigning a numerical value for stage of estrus (Diestrus = 0, Proestrus = 1, Estrus = 2, Metestrus = 3) for each of the four days of the first binge. To determine if stage of estrus affected cell survival, a factorial ANOVA using the variables Diet, Activity, and a dummy coded variable for the stage of estrus on the seventh day after the last dose of ethanol (Day of reactive proliferative burst) was analyzed.

## Results

### A second binge exposure decreased intoxication but increased withdrawal severity

**Figure 2:**
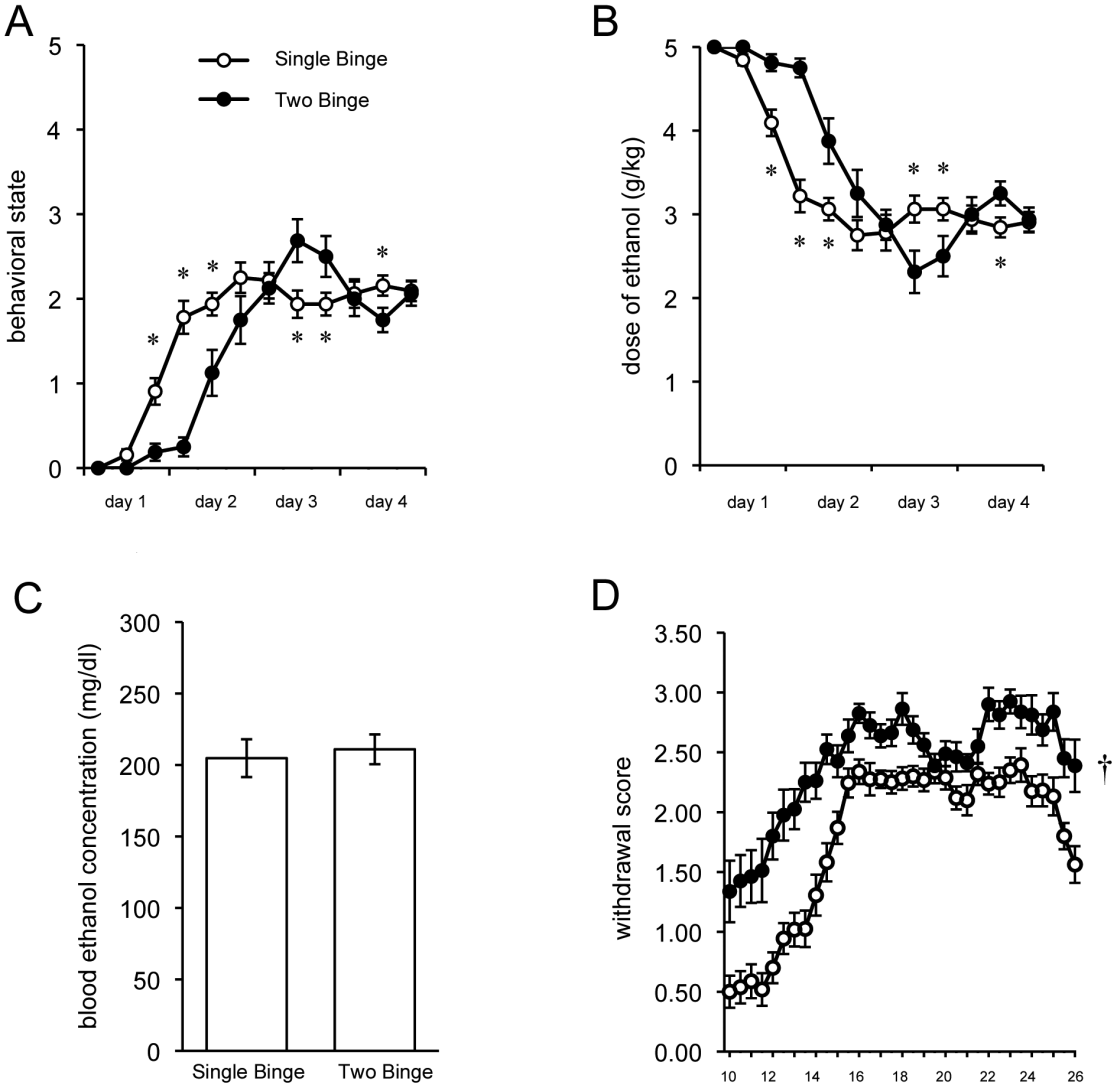
After a second binge, animals acted less intoxicated, but experienced more severe withdrawal. In this figure, “Single Binge” indicates data from animals that underwent one binge combined with the first binge data from animals that underwent 2 binges (“Two Binge”). During the second binge, animals took longer to act intoxicated (A), despite initially receiving more ethanol (B) and having blood ethanol concentrations that were similar to Single Binge (C). Following a second binge, withdrawal symptoms were significantly increased overall (D). *p<0.05; † p<0.05 significant main effect of Binge.

Animals that underwent two binge exposures acted significantly less intoxicated during the early portion of the second binge, compared to the first (repeated-measures ANOVA, significant main effect of Binge [*F*(1,46) = 10.99, *p*<0.01]) (see Figure 2A). An independent groups *t*-test comparing overall mean intoxication revealed that animals were significantly more intoxicated during the first binge [*t*(46) = 3.315, *p*<0.05]. However, peak intoxication scores did not differ between the two binges [*t*(46) = .102, *p* = 0.919]. Although they acted less intoxicated during the second binge, animals actually received more ethanol (repeated-measures ANOVA significant main effect of Binge [*F*(1,46) = 10.99, *p*<0.01] see Figure 2B). Furthermore, there was a significant main effect of Time [*F*(11,506) = 48.540, *p*<0.001] and a significant Binge×Time interaction [*F*(11,506) = 6.136, *p*<0.001]. There was a significantly greater mean dose of ethanol per day for Two Binge animals compared to all binged animals during the first binge [*t*(46) = −3.315, *p* <0.05]. However, despite Two Binge animals receiving more ethanol during the second binge, there was no difference in blood ethanol concentration between Single Binge and Two Binge animals [*t*(45) = −.312, *p* = 0.757] (see Figure 2C).

Despite acting less intoxicated during the second binge, animals had more severe withdrawal symptoms (repeated-measures ANOVA main effect of Binge [*F*(1,46) = 60.194, *p*<0.001], see Figure 2D). Additionally, a main effect of Time [(*F*(32,1472) = 32.415, *p*<0.001] and significant Binge×Time interaction [*F*(32,1472) = 2.297, *p*<0.001] indicate that the spontaneous withdrawal symptoms became more severe over time, until diminishing slightly near the end of the observation period. Average withdrawal score and peak withdrawal severity were both higher in animals experiencing a second withdrawal ([*t*(46) = −7.734, *p*<0.001] and [*t*(44.108) = −4.693, *p*<0.001], respectively). For both the Single and Two Binge animals, mean intoxication was significantly correlated with the mean withdrawal (*r^2^* = .617, *p<*.001; *r^2^* = .442, *p<*0.05).

A second binge also had an effect on body weight. At the start of the experiment, there was no difference in body weight between groups. Two-way repeated measures ANOVA across the experiment using the variables Time, Activity, Diet and their interactions revealed significant main effects of Time [*F*(38, 1596) = 675.27, *p*<0.001], Diet [*F*(2, 42) = 8.62, *p*<0.001], and a significant Time×Diet interaction [F(76, 1596) = 29.8, *p*<0.001] but no significant main effect of Activity [*F*(1, 42) = 0.140, *p* = 0.710] or Time×Activity interaction [*F*(38, 1596) = 1.5, *p* = 0.207]. This indicates that animals continued to gain weight across the course of the experiment with a difference depending on diet, with no effect of exercise on body weight. Post hoc analysis revealed that there was no difference in body weight between Control (S0 and E0) and Single Binge (S1 and E1) animals, but that a second binge transiently decreased body weight.

### Binge ethanol exposure caused enduring hippocampal damage, which was reversed by exercise

**Figure 3:**
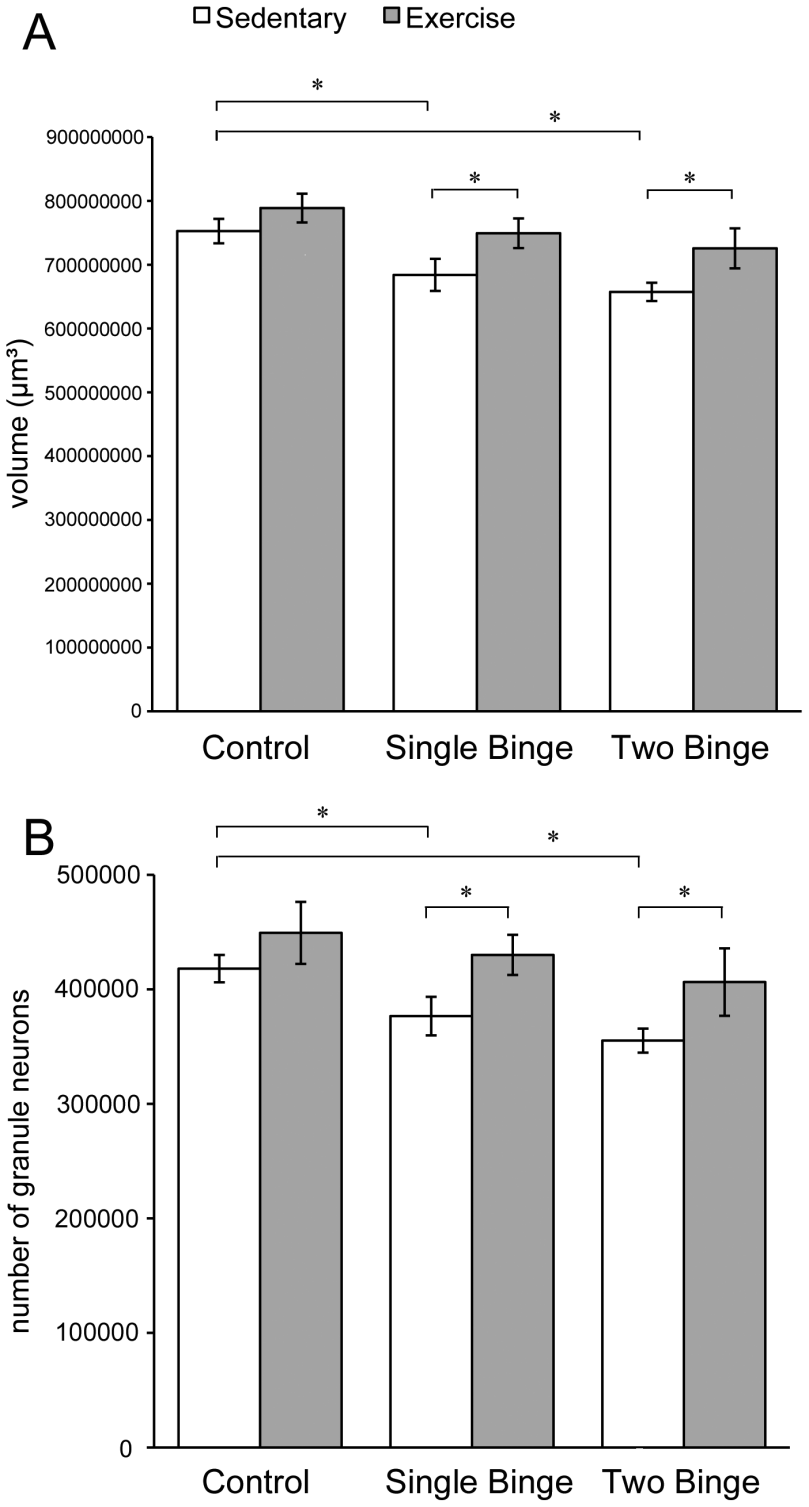
Exercise reversed binge-induced hippocampal damage. Both sedentary Single and sedentary Two Binge animals showed a significant reduction in volume of the dentate gyrus (A) and number of remaining granule neurons (B) 35 days after the end of the last binge. Post-binge exercise completely reversed these losses. * p<0.05

Binge ethanol exposure was associated with a decrease in both volume of the DG and number of granule neurons. For volume of the dentate gyrus, two-way ANOVA revealed a significant main effect of Diet [*F*(2,42) = 6.07, *p*<0.05]. Post hoc analysis showed that DG volume was smaller in both sedentary Single and sedentary Two Binge animals, compared to controls (see Figure 3A), but a second binge did not lead to a significant further volume decrease. Exercise restored DG volume (two-way ANOVA, significant main effect of Activity [*F*(1,42) = 8.92, *p*<0.05]). Post hoc comparisons showed that both exercised Single Binge and exercised Two Binge animals had larger DG volumes than their sedentary counterparts. Exercise did not, however, increase DG volume in control animals.

We found a similar pattern of results for number of DG granule neurons. Binge exposure led to a decrease in granule neurons (two-way ANOVA, significant main effect of Diet [*F*(2,42) = 10.605, *p*<0.05]). Again, both sedentary Single and sedentary Two Binge animals had fewer DG granule neurons compared with controls (see Figure 3B), but there was no significant further decrease due to a second binge. Exercise ameliorated the effect of binge exposure (two-way ANOVA significant main effect of Activity [*F*(1,42) = 23.144, *p*<0.05]). Both exercised Single and exercised Two Binge animals had significantly more DG granule neurons compared to their sedentary counterparts. The difference in number of granule neurons between exercised and sedentary control animals fell just short of statistical significance (p = 0.062).

Prior binge exposure did not affect how far animals ran during daily exercise. Repeated measures ANOVA revealed no main effect of Diet on distance run [*F*(2,21) = .783, *p* = 0.470] and no significant Diet×Time interaction [*F*(54,567) = .962, *p* = 0.555]. There was a significant main effect of Time, indicating that animals increased the distance covered during the four weeks of the exercise period [*F*(27,567) = 17.317, *p*<0.001].

### Exercise enhanced survival of cells generated during abstinence

**Figure 4:**
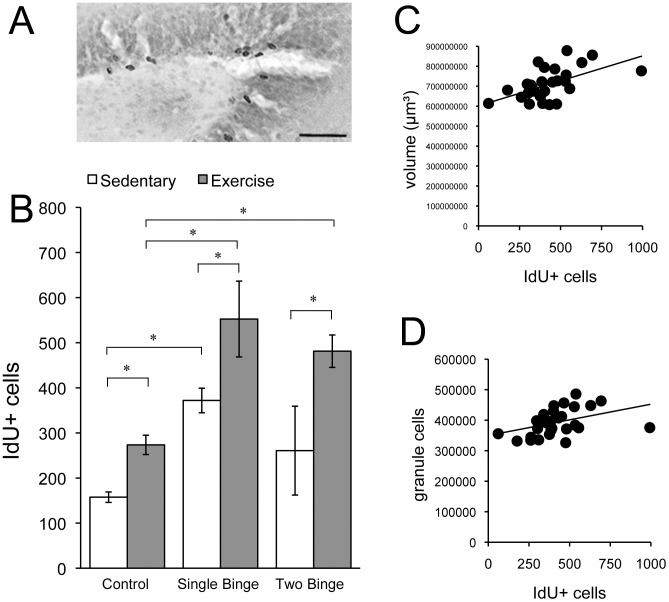
Exercise enhanced survival of cells generated post-binge. Sedentary single Binge animals had significantly more IdU+ cells (A; scale bar = 100 µm) 35 days post-binge (B). Sedentary Two Binge animals showed a non-significant increase. In all groups, exercise significantly increased the number of IdU+ cells. Within binged animals, there was a significant positive correlation between number of IdU+ cells and volume of the DG (C) and between number of IdU+ cells and number of granule neurons (D). * p<0.05

Using this binge model, a burst of cell proliferation on the seventh day of abstinence has previously been reported [18], [20]. Therefore, in the present study, we injected animals with IdU 7 days following the first binge, and then sacrificed them 28 days later (38 days later for Two Binge animals) in order to investigate the effects of exercise and/or a subsequent binge on cell survival. Brains from two animals (1 sedentary Two Binge, 1 exercise Single Binge) showed no IdU labeling, indicating faulty injection, and were excluded from this analysis. We found that binge exposure increased the number of IdU+ cells (two-way ANOVA, significant main effect of Diet [F(2,40) = 18.807, *p*<0.001], see Figure 4B). Planned post hoc comparisons showed that sedentary Single Binge (but not sedentary Two Binge) animals had significantly more IdU+ cells than sedentary controls.

Exercise increased IdU labeling (two-way ANOVA, significant main effect of Activity [F(1,40) = 26.46, *p*<0.001], see Figure 4B). Post hoc comparisons showed that there were significantly more IdU+ cells in exercised animals compared to their sedentary counterparts. Two-way ANOVA did not reveal a significant Diet×Activity interaction, indicating a similar pattern of effects of these two variables in all groups. Thus, both binge exposure and exercise enhanced IdU labeling, such that the largest effect was present in binged animals that exercised (both exercised Single and exercised Two Binge animals had significantly more IdU+ cells than exercised controls). There was no significant difference in IdU+ cells between exercised Single and exercised Two Binge animals. Within binge-exposed animals, there was a significant positive correlation between the number of IdU+ cells and volume of the DG (*r^2^* = .56, *p*<0.001) (see Figure 4C), and between number of IdU+ cells and number of granule neurons (*r^2^* = .41, *p*<0.01) (see Figure 4D).

### A second binge did not cause further hippocampal damage

It has been suggested that the repeated cycles of intoxication and abstinence that characterize binge drinking may be particularly damaging to the brain [21]–[23]. We reasoned that binge-induced self-repair processes in the hippocampus could be disrupted by subsequent binge exposures. We tested this idea in the present study by exposing animals to a second binge timed to occur during the period when self-repair processes would be expected to be underway following the first binge. Because binge ethanol exposure decreases cell proliferation in the DG [17], we expected that a second binge would decrease the number of IdU+ cells compared to animals that experienced only one binge. However, there was no significant difference in IdU+ cells between animals that experienced one binge, and animals that experienced two (*p = *.196; see Figure 4B).

**Figure 5:**
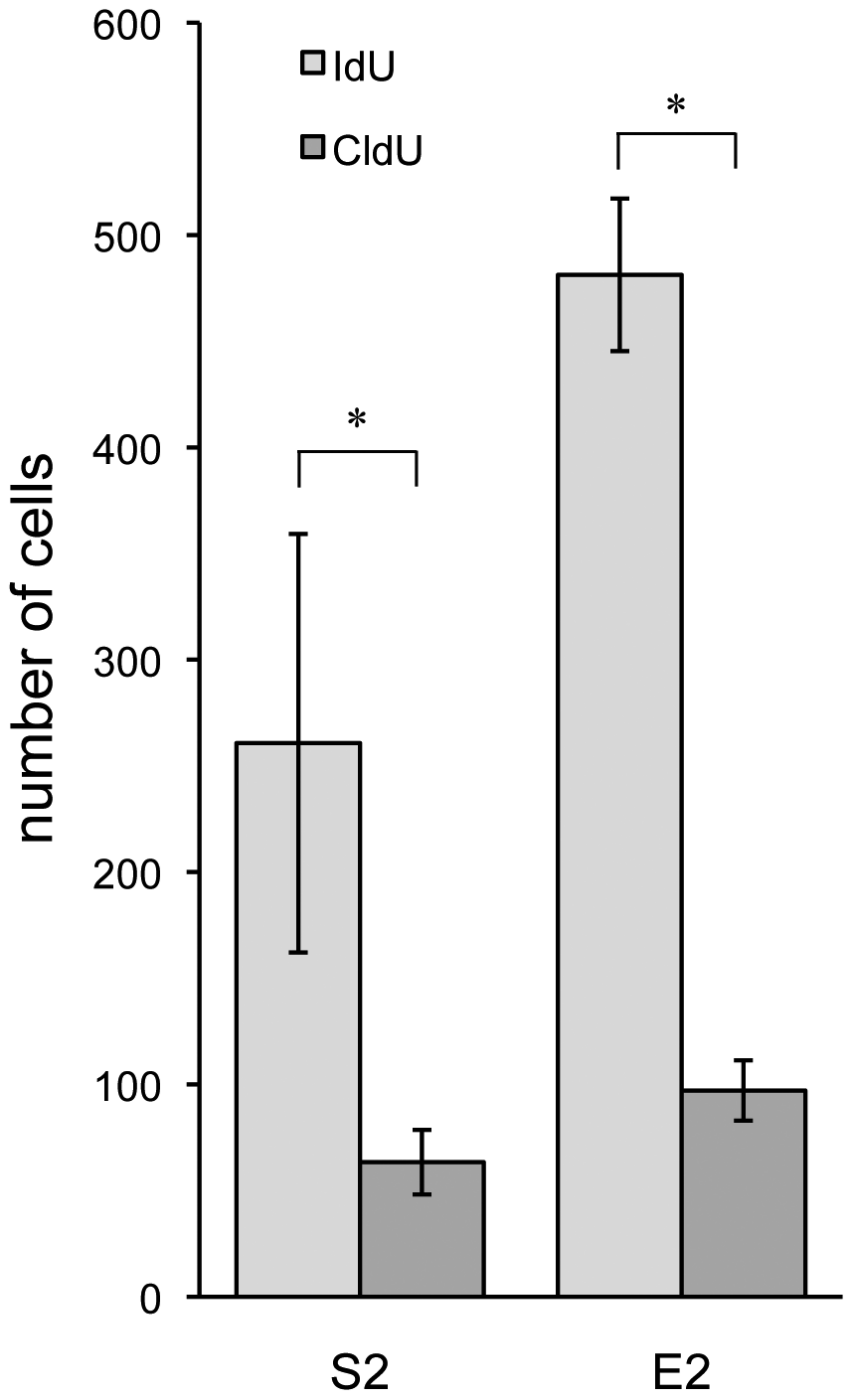
Fewer cells were labeled following a second binge. Cells generated 7 days after the first binge were labeled with IdU, while cells generated 7 days after the second binge were labeled with CldU. In both sedentary (S2) and exercised (E2) Two Binge animals, there were significantly fewer CldU+ cells, compared with IdU+ cells. *p<0.05

CldU was administered to Two Binge animals in order to label cells generated in response to the second binge exposure. An independent groups t-test revealed no significant difference [*t*(14) = −1.454, *p* = 0.790] between exercised and sedentary Two Binge animals. Within Two Binge animals (S2, E2), survival of cells generated after the second binge (CldU+ cells) was compared to survival of those generated after the first binge (IdU+ cells). Paired groups t-test revealed significantly more IdU+ cells than CldU+ cells in both sedentary [*t*(6) = 3.46, *p*<0.05] and exercised Two Binge animals [*t*(7) = 14.37, *p*<0.05] (see Figure 5).

### Stage of estrus during binge did not affect neural outcome

Previous research has shown that levels of progesterone and estrogen improve outcome in experimental models of brain injury for female rats [38]. The estrus cycle can also acutely influence cell genesis, with the highest number of new cells born during proestrus [39]. We therefore investigated whether stage of estrus on any given day of the four-day binge had a significant effect on the number of remaining granule cells or cell survival 35 days after the last dose of ethanol using a two-way ANOVA. Stage of estrus was dummy coded for each of the four days of the first binge (Diestrus = 0, Proestrus = 1, Estrus = 2, Metestrus = 3), with Diet and Activity also included in the model, and the total number of granule cells used as an outcome. For each day of the four-day binge there was a fairly even distribution of animals in each phase, although diestrus always had the largest number of animals (which is to be expected because this phase lasts the longest [40]). Two-way ANOVA revealed a significant main effect of Diet [*F*(2,12) = 9.755, *p* = 0.003] and Activity [*F*(1,12) = 8.475, *p* = 0.013] however no significant effect of stage of estrus for any of the four days of the first binge, (D1: *p* = 0.722; D2: *p* = 0.086; D3: *p* = 0.090; D4: *p* = 0.923). A separate two-way ANOVA using IdU+ cells as an outcome measure revealed a significant main effect of Diet [*F*(2, 25) = 16.283, *p*<0.05] and a significant effect of Activity [*F*(1, 25) = 18.903, *p*<0.05] but no significant effect of stage of estrus on the seventh day after the last dose of alcohol, [*F*(3,25) = .850, *p* = 0.480]. These results indicate that the stage of estrus during any day of the first binge or on the day of the reactive proliferative cell burst after a single binge exposure had no significant effect on the number of granule cells in the dentate gyrus or cell survival 35 days after the last dose of ethanol. While this does not rule out possible interactive effects of ethanol and hormone levels, it indicates that stage of estrus was not a driving factor in determining hippocampal damage in the present study.

## Discussion

### Exercise reverses lasting binge-induced hippocampal damage by enhancing survival of cells generated during abstinence

The major findings of this study are that a single binge exposure results in enduring damage to the hippocampus, and that post-binge exercise is neurorestorative. Brain damage and degeneration have previously been documented in the hippocampus and surrounding regions at acute time points using this model [13], [14], [41]. Furthermore, we have previously shown a 10–15% loss of granule neurons in the DG immediately following a binge [19]. In the present study, we show that this loss is enduring in female rats, unless they exercise after binge exposure. We found that animals that exercised post-binge had DG volumes and numbers of granule neurons similar to those of controls. Although it re-established baseline conditions, exercise did not increase the volume of the DG or the number of granule neurons in control animals, upholding the idea that brain damage creates an environment conducive to plasticity [25].

The results of the present study indicate that exercise exerts this restorative effect by augmenting endogenous self-repair. It has previously been shown that the DG reacts to the damaging effects of binge ethanol exposure by mounting two bursts of reactive cell proliferation during the first week of abstinence [18], and that many of those newly generated cells later differentiate into neurons [18], [20]. In the present study, we labeled cells generated during abstinence using IdU, and quantified their survival. Similar to previous reports [18], [20], we found increased numbers of labeled cells in sedentary binged animals, indicating that the hippocampus responded to binge exposure by increasing cell genesis. We found a further increase in labeled cells in animals that exercised. In addition, we found significant correlations between IdU-labeled cells and the volume of the granular layer and between IdU-labeled cells and number of granule neurons. Taken together, these data indicate that post-binge exercise capitalizes on the increased cell proliferation that naturally occurs after binge exposure, thereby enabling complete repopulation of the DG granular layer.

The potential mechanisms underlying the augmentation of cell survival are many. It may be that in sedentary animals there is insufficient trophic support to repair existing cells as well as promote the development of those generated post-binge. Established cells may be able to command the bulk of available trophic support, leaving insufficient levels to nurture the development of newly generated cells. Exercise, which powerfully enhances neurotrophin availability [42]–[48], may be capable of ensuring a sufficient supply of trophic molecules, so that both mature and newly generated cells are supported. We will direct future efforts towards the study of exercise-induced increases in available trophic support in the alcohol-damaged brain.

### The hippocampus may respond differently to a second binge

Prior work using this model, as well as results of the present study, indicates that there is a significant increase in cell genesis seven days post-binge. In the present study, we investigated what would happen to post-binge cell genesis if a second binge exposure occurred. We reasoned that since cell proliferation is decreased by binge exposure [17], a second binge would suppress reactive cell genesis. We therefore expected to see fewer IdU+ cells in Two Binge animals, compared to Single Binge animals. Contrary to our hypothesis, we found no difference in IdU+ cells between sedentary Two Binge and sedentary Single Binge animals. There was, however, considerable variability in the number of IdU+ cells in the sedentary Two Binge group, suggesting that a second binge did suppress cell proliferation in some animals.

We also wanted to see if the number of cells generated in response to a second binge would differ from the number generated after the first binge. To investigate this, we administered CldU to rats 7 days following a second binge exposure. We then compared the number of CldU+ cells (those generated after the second binge) to the number of IdU+ cells (those generated after the first binge). We found that there were significantly fewer CldU+ cells (see Figure 5). There are several possible explanations for this finding. First, it may be that there was a robust response to a second binge, but that it occurred more or less rapidly (ie, before or after day 7). Second, it is possible that, having mounted a robust response to the first binge, the brain is unable to mount another one and that the magnitude of the second reaction was diminished. However, the most likely explanation is that a second binge caused no significant further damage to the hippocampus, rendering a large response unnecessary.

It is interesting to note that there was no further increase in hippocampal damage (as determined by volume of the DG and number of remaining granule neurons) in animals that underwent a second binge. This finding is surprising, given that a binge-drinking pattern is associated with significant neural damage in humans [3], [5], [49]. However, prior studies of this model indicate that the majority of damage is occurring during the binge itself, and not during or after withdrawal [15]. Additionally, we have previously shown a 10–15% decrement in granule neuron numbers immediately after a binge (before onset of withdrawal), thereby further supporting the idea that most of the damage is done during intoxication. In the present study, we found that animals acted less intoxicated during a second binge exposure, despite receiving more ethanol and having BECs not different from animals during the first binge. The decreased intoxication may be a behavioral manifestation of compensatory changes in the brain that enable it to better withstand ethanol. In other words, we have behavioral evidence in this study that, during a second binge, the brain was better equipped to handle a high BEC, and thus perhaps better able to defend itself against mechanisms of damage occurring during the binge. If this is true, then timing a second binge after a longer period of abstinence may result in more hippocampal damage.

## Conclusions

In the present study, we found that binge ethanol exposure results in lasting hippocampal damage in female rats, and that this damage was completely reversed by post-binge exercise. This restorative effect is apparently due to augmentation of endogenous self-repair processes. Our results suggest that exercise may be an effective means by which to enhance neural recovery after alcohol-induced damage.

## References

[1] CDC (2012) Vital signs: Binge drinking prevalence, frequency and intensity among adults - United States, 2010. MMWR Surveill Summ 61: 14–19. 22237031

[2] Crews FT, Boettiger CA (2009) Impulsivity, frontal lobes and risk for addiction. Pharmacol Biochem Behav 93: 237–247. 19410598 10.1016/j.pbb.2009.04.018 PMC2730661

[3] Duka T, Trick L, Nikolaou K, Gray MA, Kempton MJ, et al (2011) Unique Brain Areas Associated with Abstinence Control Are Damaged in Multiply Detoxified Alcoholics. Biol Psychiatry 70: 545–552. 21612768 10.1016/j.biopsych.2011.04.006 PMC3165202

[4] Bechara A (2005) Decision making, impulse control and loss of willpower to resist drugs: a neurocognitive perspective. Nat Neurosci 8: 1458–1463. 16251988 10.1038/nn1584

[5] Duka T, Townshend JM, Collier K, Stephens DN (2003) Impairment in cognitive functions after multiple detoxifications in alcoholic inpatients. Alcohol Clin Exp Res 27: 1563–1572. 14574226 10.1097/01.ALC.0000090142.11260.D7

[6] Sundell L, Salomaa V, Vartiainen E, Poikolainen K, Laatikainen T (2008) Increased stroke risk is related to a binge-drinking habit. Stroke 39: 3179–3184. 18832741 10.1161/STROKEAHA.108.520817

[7] Jarvenpaa T, Rinne JO, Koskenvuo M, Raiha I, Kaprio J (2005) Binge drinking in midlife and dementia risk. Epidemiology 16: 766–771. 16222166 10.1097/01.ede.0000181307.30826.6c

[8] Gupta S, Warner J (2008) Alcohol-related dementia: a 21st-century silent epidemic? Br J Psychiatry 193: 351–353. 18978310 10.1192/bjp.bp.108.051425

[9] Pfefferbaum A, Sullivan EV, Mathalon DH, Shear PK, Rosenbloom MJ, et al (1995) Longitudinal changes in magnetic resonance imaging brain volumes in abstinent and relapsed alcoholics. Alcohol Clin Exp Res 19: 1177–1191. 8561288 10.1111/j.1530-0277.1995.tb01598.x

[10] Agartz I, Brag S, Franck J, Hammarberg A, Okugawa G, et al (2003) MR volumetry during acute alcohol withdrawal and abstinence: a descriptive study. Alcohol Alcohol 38: 71–78. 12554612 10.1093/alcalc/agg020

[11] Cardenas VA, Studholme C, Gazdzinski S, Durazzo TC, Meyerhoff DJ (2007) Deformation-based morphometry of brain changes in alcohol dependence and abstinence. Neuroimage 34: 879–887. 17127079 10.1016/j.neuroimage.2006.10.015 PMC1865510

[12] Wobrock T, Falkai P, Schneider-Axmann T, Frommann N, Wolwer W, et al (2009) Effects of abstinence on brain morphology in alcoholism: a MRI study. Eur Arch Psychiatry Clin Neurosci 259: 143–150. 19165528 10.1007/s00406-008-0846-3 PMC3085767

[13] Obernier JA, Bouldin TW, Crews FT (2002) Binge ethanol exposure in adult rats causes necrotic cell death. Alcohol Clin Exp Res 26: 547–557. 11981132

[14] Obernier JA, White AM, Swartzwelder HS, Crews FT (2002) Cognitive deficits and CNS damage after a 4-day binge ethanol exposure in rats. Pharmacol Biochem Behav 72: 521–532. 12175448 10.1016/s0091-3057(02)00715-3

[15] Crews FT, Nixon K (2009) Mechanisms of neurodegeneration and regeneration in alcoholism. Alcohol Alcohol 44: 115–127. 18940959 10.1093/alcalc/agn079 PMC2948812

[16] Collins MA, Corso TD, Neafsey EJ (1996) Neuronal degeneration in rat cerebrocortical and olfactory regions during subchronic "binge" intoxication with ethanol: possible explanation for olfactory deficits in alcoholics. Alcohol Clin Exp Res 20: 284–292. 8730219 10.1111/j.1530-0277.1996.tb01641.x

[17] Nixon K, Crews FT (2002) Binge ethanol exposure decreases neurogenesis in adult rat hippocampus. J Neurochem 83: 1087–1093. 12437579 10.1046/j.1471-4159.2002.01214.x

[18] Nixon K, Crews FT (2004) Temporally specific burst in cell proliferation increases hippocampal neurogenesis in protracted abstinence from alcohol. J Neurosci 24: 9714–9722. 15509760 10.1523/JNEUROSCI.3063-04.2004 PMC6730141

[19] Leasure JL, Nixon K (2010) Exercise neuroprotection in a rat model of binge alcohol consumption. Alcohol Clin Exp Res 34: 404–414. 20028365 10.1111/j.1530-0277.2009.01105.x PMC2936244

[20] Nixon K, Kim DH, Potts EN, He J, Crews FT (2008) Distinct cell proliferation events during abstinence after alcohol dependence: microglia proliferation precedes neurogenesis. Neurobiol Dis 31: 218–229. 18585922 10.1016/j.nbd.2008.04.009 PMC2680247

[21] Stephens DN, Duka T (2008) Review. Cognitive and emotional consequences of binge drinking: role of amygdala and prefrontal cortex. Philos Trans R Soc Lond B Biol Sci 363: 3169–3179. 18640918 10.1098/rstb.2008.0097 PMC2607328

[22] Hunt WA (1993) Are binge drinkers more at risk of developing brain damage? Alcohol 10: 559–561. 8123218 10.1016/0741-8329(93)90083-z

[23] Tivis R, Beatty WW, Nixon SJ, Parsons OA (1995) Patterns of cognitive impairment among alcoholics: are there subtypes? Alcohol Clin Exp Res 19: 496–500. 7625588 10.1111/j.1530-0277.1995.tb01537.x

[24] Kozlowski DA, Leasure JL, Schallert T (2013) The control of movement following traumatic brain injury. Compr Physiol 3: 121–139. 23720282 10.1002/cphy.c110005

[25] Schallert T, Jones TA (1993) "Exuberant" neuronal growth after brain damage in adult rats: the essential role of behavioral experience. J Neural Transplant Plast 4: 193–198. 8018751 10.1155/NP.1993.193 PMC2565265

[26] Schallert T, Kozlowski DA, Humm JL, Cocke RR (1997) Use-dependent structural events in recovery of function. Adv Neurol 73: 229–238. 8959217

[27] Will B, Galani R, Kelche C, Rosenzweig MR (2004) Recovery from brain injury in animals: relative efficacy of environmental enrichment, physical exercise or formal training (1990–2002). Prog Neurobiol 72: 167–182. 15130708 10.1016/j.pneurobio.2004.03.001

[28] van Praag H, Christie BR, Sejnowski TJ, Gage FH (1999) Running enhances neurogenesis, learning, and long-term potentiation in mice. Proc Natl Acad Sci U S A 96: 13427–13431. 10557337 10.1073/pnas.96.23.13427 PMC23964

[29] van Praag H, Kempermann G, Gage FH (1999) Running increases cell proliferation and neurogenesis in the adult mouse dentate gyrus. Nat Neurosci 2: 266–270. 10195220 10.1038/6368

[30] Helfer JL, Goodlett CR, Greenough WT, Klintsova AY (2009) The effects of exercise on adolescent hippocampal neurogenesis in a rat model of binge alcohol exposure during the brain growth spurt. Brain Res 1294: 1–11. 19647724 10.1016/j.brainres.2009.07.090 PMC2756176

[31] Redila VA, Olson AK, Swann SE, Mohades G, Webber AJ, et al (2006) Hippocampal cell proliferation is reduced following prenatal ethanol exposure but can be rescued with voluntary exercise. Hippocampus 16: 305–311. 16425237 10.1002/hipo.20164

[32] Thomas JD, Sather TM, Whinery LA (2008) Voluntary exercise influences behavioral development in rats exposed to alcohol during the neonatal brain growth spurt. Behav Neurosci 122: 1264–1273. 19045946 10.1037/a0013271 PMC3164868

[33] Majchrowicz E (1975) Induction of physical dependence upon ethanol and the associated behavioral changes in rats. Psychopharmacologia 43: 245–254. 1237914 10.1007/BF00429258

[34] Penland S, Hoplight B, Obernier J, Crews FT (2001) Effects of nicotine on ethanol dependence and brain damage. Alcohol 24: 45–54. 11524181 10.1016/s0741-8329(01)00142-2

[35] Humm JL, Kozlowski DA, James DC, Gotts JE, Schallert T (1998) Use-dependent exacerbation of brain damage occurs during an early post- lesion vulnerable period. Brain Res 783: 286–292. 9507166 10.1016/s0006-8993(97)01356-5

[36] Griesbach GS, Gomez-Pinilla F, Hovda DA (2004) The upregulation of plasticity-related proteins following TBI is disrupted with acute voluntary exercise. Brain Res 1016: 154–162. 15246851 10.1016/j.brainres.2004.04.079

[37] Paxinos G, Watson C (1998) The Rat Brain in Stereotaxic Coordinates. San Diego: Academic Press.

[38] Roof RL, Duvdevani R, Stein DG (1993) Gender influences outcome of brain injury: progesterone plays a protective role. Brain Res 607: 333–336. 8481809 10.1016/0006-8993(93)91526-x

[39] Tanapat P, Hastings NB, Reeves AJ, Gould E (1999) Estrogen stimulates a transient increase in the number of new neurons in the dentate gyrus of the adult female rat. J Neurosci 19: 5792–5801. 10407020 10.1523/JNEUROSCI.19-14-05792.1999 PMC6783062

[40] Goldman JM, Murr AS, Cooper RL (2007) The rodent estrous cycle: characterization of vaginal cytology and its utility in toxicological studies. Birth Defects Res B Dev Reprod Toxicol 80: 84–97. 17342777 10.1002/bdrb.20106

[41] Kelso ML, Liput DJ, Eaves DW, Nixon K (2011) Upregulated vimentin suggests new areas of neurodegeneration in a model of an alcohol use disorder. Neuroscience 197: 381–393. 21958862 10.1016/j.neuroscience.2011.09.019 PMC3298440

[42] Gomez-Pinilla F, So V, Kesslak JP (1998) Spatial learning and physical activity contribute to the induction of fibroblast growth factor: neural substrates for increased cognition associated with exercise. Neuroscience 85: 53–61. 9607702 10.1016/s0306-4522(97)00576-9

[43] Gomez-Pinilla F, Ying Z, Opazo P, Roy RR, Edgerton VR (2001) Differential regulation by exercise of BDNF and NT-3 in rat spinal cord and skeletal muscle. European Journal of Neuroscience 13: 1078–1084. 11285004 10.1046/j.0953-816x.2001.01484.x

[44] Gomez-Pinilla F, Dao L, So V (1997) Physical exercise induces FGF-2 and its mRNA in the hippocampus. Brain Res 764: 1–8. 9295187 10.1016/s0006-8993(97)00375-2

[45] Gomez-Pinilla F, Vaynman S, Ying Z (2008) Brain-derived neurotrophic factor functions as a metabotrophin to mediate the effects of exercise on cognition. Eur J Neurosci 28: 2278–2287. 19046371 10.1111/j.1460-9568.2008.06524.x PMC2805663

[46] Berchtold NC, Chinn G, Chou M, Kesslak JP, Cotman CW (2005) Exercise primes a molecular memory for brain-derived neurotrophic factor protein induction in the rat hippocampus. Neuroscience 133: 853–861. 15896913 10.1016/j.neuroscience.2005.03.026

[47] Berchtold NC, Kesslak JP, Pike CJ, Adlard PA, Cotman CW (2001) Estrogen and exercise interact to regulate brain-derived neurotrophic factor mRNA and protein expression in the hippocampus. Eur J Neurosci 14: 1992–2002. 11860494 10.1046/j.0953-816x.2001.01825.x

[48] Trejo JL, Carro E, Nunez A, Torres-Aleman I (2002) Sedentary life impairs self-reparative processes in the brain: the role of serum insulin-like growth factor-I. Rev Neurosci 13: 365–374. 12542262 10.1515/revneuro.2002.13.4.365

[49] Duka T, Gentry J, Malcolm R, Ripley TL, Borlikova G, et al (2004) Consequences of multiple withdrawals from alcohol. Alcohol Clin Exp Res 28: 233–246. 15112931 10.1097/01.alc.0000113780.41701.81

